# Development and psychometric evaluation of a questionnaire to measure cancer patients’ perception of care coordination

**DOI:** 10.1186/s12913-020-4905-4

**Published:** 2020-01-21

**Authors:** Izumi Okado, Kevin Cassel, Ian Pagano, Randall F. Holcombe

**Affiliations:** University of Hawai‘i Cancer Center, 701 Ilalo St. 6th Floor, Honolulu, HI 96813 USA

**Keywords:** Care coordination, Cancer, Instrument, Questionnaire, Psychometric evaluation

## Abstract

**Background:**

Although the importance of care coordination (CC) is well-recognized, cancer patients often receive poorly coordinated care across varied care settings and different oncology providers. Efforts to improve cancer care are hampered by lack of adequate measures. In this two-part, mixed-method study, we describe the development, refinement, and validation of a new care coordination instrument (CCI) designed to assess cancer patients’ perception of CC.

**Methods:**

In Study 1, an initial CCI was developed incorporating questions based on literature review. The items were then modified following four field tests conducted in a large academic hospital with oncology nurses (*n* = 20) and cancer patients (*n* = 120). This modified instrument was used to determine whether the CCI was able to distinguish CC between two practices (30 GI and 30 myeloma patients) within the same hospital setting. In Study 2, 68 patients receiving community-based care participated in seven focus groups. Based on these discussions, the CCI items were again refined, and psychometric evaluation was conducted to assess the quality of the instrument.

**Results:**

Based on field tests, 3 domains of the CCI, Communication, Navigation, and Operational, were defined as critical components of CC. The Operational domain evaluates efficiency of care and is unique to this CCI. The field test demonstrated that GI patients reported significantly better CC Overall and for the Communication and Navigation domains (all *p* < .05). In Study 2, patients expressed concordance with the CCI items and their CC experiences, establishing validity of the CCI. Qualitative analysis of the focus group discussions indicated that the items with the highest frequencies of participants’ comments were related to the concepts of Navigator, Team, Survey, and Communication. Quantitative analysis identified items with a limited response range or high rates of “neutral” responses; accordingly, those items were removed. The final CCI survey is a 29 item, multiple-choice questionnaire with excellent reliability, Cronbach’s α = .922.

**Conclusions:**

We developed a novel, patient-centered tool with excellent psychometric properties that can be utilized across varied practice settings to assess patients’ perception of cancer care coordination.

**Trial registration:**

Not required; retrospectively registered ClinicalTrials.gov NCT03594006 20 July 2018.

## Background

Care coordination (CC) is a complex concept that greatly influences a patient’s perception of his/her interaction with a healthcare delivery system and can directly and indirectly affect the quality of patient care. Although the importance of CC is well-recognized, existing evidence demonstrates that cancer patients often receive poor, fragmented care across multiple settings and providers [1–4]. Poorly coordinated care has been linked to numerous adverse outcomes including medical errors, patient dissatisfaction, higher health care costs, excessive use of health services, and increased morbidity and mortality from the disease [5–8]. Further, the critical need to improve the quality and value of health care delivery has been highlighted by a recent report that demonstrated that failure in care coordination costs $27.2 to $78.2 billion annually in the U.S. health care system [9]. To date, efforts to improve cancer care delivery have been hampered by the lack of adequate patient-centered measures to assess cancer care coordination. Given the substantial costs and potentially detrimental impacts of poorly coordinated care for cancer patients [5], it is critical to address these measurement gaps in order to further our understanding of current CC approaches and to identify factors that contribute to effective versus ineffective cancer care coordination.

The Agency for Healthcare Research and Quality (AHRQ) defines CC as “deliberate organization of patient care activities between two or more participants (including the patient) involved in a patient’s care to facilitate the appropriate delivery of health care services” [1]. Although care coordination involves multiple participants, cancer patients and their families often serve as care coordinators, arranging appointments, seeking and furnishing information, and navigating the many steps in care [10]. Important for all patients, care coordination is especially critical for cancer patients as therapeutic interventions involve multiple episodes of care, numerous healthcare providers, varied health care settings, and a high overall symptom burden [11]. Effective CC is a key component of cancer care delivery, and provisions of well-coordinated care increase the efficiency of health care delivery and improve patient outcomes [5]. Accordingly, a coordinated, team-based, patient-centered approach to care has been highlighted by the Institute of Medicine (IOM) as a hallmark of a high-quality cancer care delivery system [3].

In response to efforts to improve the quality of care, research on cancer care delivery has rapidly expanded in recent years. A growing body of evidence has provided some insights on cancer CC, mostly based on health care systems or providers’ perspectives on CC. According to research conducted within closed systems such as the Veterans Administration Health System and Kaiser, patients have generally reported favorable cancer care coordination experiences [10, 12–14]. It is possible that positive reports of cancer care coordination experiences in these closed systems are due to limited numbers of providers involved and centralized systems that allow easier access to non-provider based personnel. Other systems-level research that examined the effects of specific CC interventions such as patient navigation suggests that there are promising approaches to CC intervention [15–17]. In contrast, studies on health care providers’ reports of CC have documented that there are some potential areas of improvement such as greater availability of resources, use of multidisciplinary teams and interventions, and enhanced communication between primary care physicians and oncologists [15–19].

Despite emerging research on cancer care delivery, there is relatively little empirical research on patients’ perception of cancer CC. Limited research on patient-reports of CC generally indicates that patients identify lack of coordinated care as a substantial barrier to cancer care [20–22]. However, prior research has yielded mixed findings. It is possible that sampling and methodological differences across prior studies partly account for discrepant results. On the one hand, research focused on patients with breast cancer has generally reported positive patients’ views of CC [23, 24]. Given that breast cancer is the most commonly diagnosed cancer in women [25] and that there are more breast cancer patients participating in social support groups [26], it is likely that the availabiltiy of various support resources mitigates some of the CC-related challenges in these patients. On the other hand, studies on patients with complex cancers such as pancreatic, lung, GI, and head and neck have generally reported poorer perception of CC [27–29]. Other research suggests that poorer perception of CC may be associated with patient characteristics such as having comorbid conditions [27, 28], greater levels of depression [13], and lower levels of health literary [23, 28]. Further, poor care coordination often manifests in specific racial and ethnic population groups with disparities in cancer health outcomes [30, 31]. Taken together, the available evidence suggests that there are some patient-level characteristics that influence patients’ perception of CC; however, the processes and specific aspects of cancer CC associated with optimal versus poor CC remain relatively unknown.

Another critical barrier to improving care delivery is the gap in measures for cancer care coordination [3]. According to the AHRQ Care Coordination Measures Database and the Measures for Person Centred Coordinated Care [32, 33], many validated measures for primary and in-patient care exist, but very few are designed to evaluate patients’ perception of CC specifically in cancer patients. Moreover, prior research on CC has primarily focused on health systems or providers’ perspectives, and many are designed to measure satisfaction with care rather than CC. A patient-centered approach to care requires a standardized measurement system that captures experiences from the patient perspective as well as providing a model for implementing in clinical research studies [34, 35]. Patient-reports of care coordination may not accurately identify the nature of information exchange within their care team, but patients are able to identify specific coordination processes or activities associated with poor CC experiences such as lack of adequate information regarding next steps in care delivery or confusion during the transitions in care [10]. Although a small number of prior studies have evaluated the measurement of patients’ perspectives of CC [36–38], findings are inconsistent. In the CCCQ-P survey developed and validated in Australia, two domains of care coordination are measured [38]; in contrast, CAHPS, which is developed in the US, assesses six dimensions of cancer care that includes 3-item care coordination measure [37]. Existing CC measures have many methodological and application challenges such as lack of rigorous psychometric evaluation, limited range of applicable settings, lengthy questionnaires, and limited generalizability of the survey instrument [4, 35]. Further, these measures were generally developed and geared for use at large institutions and by specialty providers, and only a few studies have examined optimal methods to assess patients’ perspectives of CC in community settings [10]. In the United States, over 75% of patients receive community-based care, which refers to private practice or hospital settings in or near the communities where cancer patients reside [39, 40]. In contrast, smaller proportions of cancer patients receive care in academic institutions or major cancer centers that are located in largely urban areas [40]. Moreover, as indicated by a systematic review by Gorin and colleagues [5], there are substantial variations in the validity and reliability of cancer CC measures.

Taken together, findings from prior work suggest the critical need for adequate, patient-centered CC measures or tools that can be used across diverse practice settings and patient populations. Such a tool may further our understanding of current CC approaches and identify specific CC processes and activities associated with both optimal and poorly coordinated care. A validated, patient CC measure with adequate psychometric properties may facilitate quality improvement efforts by informing strategies for CC improvement and identifying aspects of care that may be specific targets for intervention to improve care delivery.

### Present study

In order to address these knowledge gaps, a new survey instrument, a Care Coordination Instrument (CCI), was developed to measure patients’ perception of cancer care coordination across varied healthcare settings and populations. Given the emphasis toward a patient-centered model of care that integrates patients’ perspectives and preferences, a measure that assesses patients’ perspectives of cancer care coordination that can be easily incorporated into existing clinic workflows and patient portals is needed to further efforts to improve cancer care delivery. To that end, we employed a comprehensive approach to develop, evaluate, and refine the CCI as a self-report questionnaire with robust psychometric properties. This instrument was derived from and expands on existing care coordination framework by the AHRQ [32] and includes three domains of care coordination conceptualized as central to patient-centered CC: Communication, Navigation, and Operational. The CCI items were designed to expand the two-main AHRQ framework in order to encompass patients’ preferences and perspectives of CC processes and goals.

The present study describes the development and validation of the CCI conducted in two phases; 1) development and field testing using mixed-methods, and 2) validation and refinement of the CCI survey questionnaire based on focus group interviews with cancer patients. A better understanding of patients’ perception of CC can inform ways to improve cancer care delivery and may benefit oncology providers, hospital administrators, and patients as we work toward developing effective and efficient care delivery models.

## Methods

### Study 1: instrument development and field testing

The draft CCI items were prepared based on a review of the literature by the research team #1. First, a systematic PubMed search was performed to identify prior work on cancer care coordination measures. Search terms were entered in the following order: 1) “care coordination”; 2) “care coordination AND cancer”, and 3) “care coordination AND cancer AND tool/measure”. Of the 1918 publications identified in the first step using the search term “care coordination”, 151 focused on cancer; among those publications, only 10 publications included the key terms “cancer care coordination”, “tool”, or “measure”. Of the 10 articles, only three referenced a specific, patient-focused care coordination measurement process or instrument. This review confirmed a major gap in this literature, as relatively little research has specifically addressed cancer care coordination measures.

Next, existing CC framework and instruments were reviewed to identify relevant terminology, concepts, and care coordination processes. Information derived from theoretical models and existing care coordination instruments designed for oncology and primary care served as a foundation for the CCI items. Existing measures that were used as starting points for the development of the CCI include the Adapted Picker Institute Cancer Survey [41], Consumer Assessment of Healthcare Providers and Systems (CAHPS) [37], and the Cancer Care Coordination Questionnaire for Patients (CCCP-Q) [36, 38]. The Adapted Picker Institute Cancer Survey is a 34-item questionnaire covering coordination of care as well as confidence in providers, treatment information, and access to care, and is designed to assess patients’ experiences with cancer care and health-related quality of life [41]. The CC section of this survey addresses multidisciplinary communication but does not include transitions in care, goals of care, navigator functions, or coordination with ancillary services. A widely used patient measure, the CAHPS, was originally developed to assess patient satisfaction, and the current CAHPS Cancer Care Survey consists of six core composite measures with 56 questions [37]. Although the CAHPS Cancer Care Survey assesses patient-reports of clinician behaviors, its 3-item Care Coordination scale has weak internal consistency reliability and the lowest reliability among the six domains assessed in this survey [37]. Given that many cancer patients receive a combination of two or more interventions, the CAHPS for Cancer Survey will not capture important components of CC related to communication between different disciplines and across varied health care settings. The CCCQ-P is an Australian measure designed to assess cancer patients’ perception of care coordination [38]. Although many constructs underlying the CCCQ-P are also relevant to cancer CC in the United States, given that there are many differences in cancer care between Australian and US health care systems including a universal health care system with a large parallel private health care system and greater provisions of cancer care by general practitioners and surgeons in Australia [42–44], generalizability of the CCCQ-P to US patients is uncertain. In light of these limitations above in existing measures, the CCI was designed to complement ongoing efforts to develop care coordination measurement as well as expand on the existing framework, with greater flexibility in application and utility across varied practice settings and diverse patient populations.

Following instrument development and IRB approval, a series of field tests were conducted at Mount Sinai Hospital, a large academic hospital in New York City. During field testing, focus group discussions and modified cognitive interviews were conducted to obtain feedback from patients and providers on the CCI items. Modified cognitive interviewing is an evidence-based, qualitative method specifically designed to test whether a survey fulfills its intended purpose, and it is commonly used before data collection for initial testing of a survey [45]. Field testing consisted of four independent samples; 1) Review by 20 oncology RNs and NPs, 2) 30 patients, 3) 30 patients, and 4) 60 patients. Each sample was tested sequentially, and between each testing the research team examined patients’ comments and modified the CCI if necessary. The fourth sample included 30 myeloma and 30 GI patients derived from the same hospital but with different specialty providers; thus, this sample served to determine whether the CCI is able to differentiate patients’ perception of CC across different practices. Adult patients (age 18 and over) with any type of cancer receiving active therapy were eligible to participate in the study. Active treatment was defined as having a minimum of three outpatient therapy or cancer care visits within a preceding 6-month period. Given that myeloma and GI patients were seen in the same hospital clinic where the overall infrastructure for clinical practices included common operational aspects such as electronic health record systems, clinic staff, and a call center, it was anticipated that only the Overall, Communication, and Navigation domains scores would differ between the two groups, and that no differences would be found for the Operational domain. Thus, this sample served as initial validation for the three CC domains of the CCI.

### Study 2: validation and refinement

In order to increase the generalizability of the CCI to community-based practices including private and hospital-based settings, a focus group study was conducted in Hawai‘i by research team #2. Prior to the first focus group interview, the readability of the CCI was evaluated using the Fleisch-Kincaid Grade Level. Analysis showed that the reading grade level was 10.1, indicating that the CCI was at a high school (10th grade) reading level, and some items may be difficult to read and understand for a general adult population. Accordingly, the structure and wording of the CCI items were examined, and the length of several questions were shortened to improve readability. Re-assessment of the reading level showed that the Fleisch-Kincaid Grade Level was 8.2, which was deemed as acceptable for the target population in this study.

Focus groups were comprised of cancer patients recruited through study flyers posted in waiting rooms at private practice and hospital-based sites, and through presentations at community support groups. Paralleling Study 1 eligibility criteria, adult patients (age 18 and over) with any type of cancer receiving active therapy were eligible to participate in Study 2. Prior to each focus group, participants were asked to review and complete a written informed consent form, a brief demographic questionnaire, and the CCI. Each focus group discussion was facilitated by two co-facilitators and conducted in a private conference room at the cancer center or a hospital. Discussions were semi-structured, with facilitators asking pre-determined questions from the discussion guide developed by the research team (see Additional file 1 for the focus group discussion guide). Open-ended questions were used to assess face and content validity of the CCI and to probe for participants’ feedback on whether any CCI items were unclear, not relevant, or needed refinement. Some of the questions in the discussion guide included “What did you think about the survey?”, “How did this survey capture your experiences with care coordination?”, and “What information related to care coordination that you think might be missing from the current survey?”. In order to assess whether the CCI adequately captured patients’ CC experiences as well as to probe for any additional aspects of CC that were not in the earlier versions of CC that may be relevant to patients receiving community-based care, additional questions on patients’ CC experiences were included in the focus group discussions. Each discussion lasted about 60–90 min, and all discussions were transcribed verbatim for analysis, with participants’ names and other identifying information (e.g., physician or other provider names mentioned during the discussions) removed to ensure anonymity. Following completion of focus groups, the research team reviewed and analyzed the transcripts, and the CCI was modified based on focus group discussions. An additional informal focus group session was conducted following the revision to ensure that the final version of the CCI demonstrated face and content validity and had acceptable readability. The University of Hawai‘i Institutional Review Board approved all study procedures and documents.

### Analyses

For Study 1, descriptive analyses (e.g., frequency distribution, means, standard deviations) were conducted at various stages of field testing to identify item(s) requiring modification or refinement. Visual inspections of response patterns served to describe mean differences and identify any atypical response patterns (e.g., skew, ceiling effect). In the fourth sample that compared patients’ responses between two oncology practices, independent-samples t-tests were conducted on overall and three domain scores. For quantitative analysis, the overall score was computed by summing responses across all the CCI items, with higher score indicating patient-reports of better CC.

For Study 2, a qualitative evaluation of the focus group transcripts was undertaken by the research team, using aspects of thematic analysis [46]. First, we openly coded narratives using an inductive approach to thematically characterize participants’ discussions that often ventured beyond the constraints of the existing CCI item domains. For initial analysis, 1–2 transcripts were coded independently by the members of the research team. After this phase, we hypothesized relationships, identified new items and reassigned existing CCI items to their respective domains as driven by the participant narratives. Each researcher identified and nominated common themes and preliminary codes, and all codes were reviewed and discussed by the team until a final consensus was reached. A codebook was subsequently developed and used to drive a subsequent thematic analysis of the additional 1–2 transcript data by each researcher. Final codes and themes were entered in NVivo v.11.4.3 for analysis. Following qualitative evaluation of focus group discussions, descriptive analyses (e.g., frequency distributions, means, standard deviations) were conducted to characterize the study sample and evaluate response patterns for each CCI item.

## Results

### Study 1: field testing

First, the draft version of the CCI was submitted to RN and NPs for review. Based on their feedback, the CCI was modified to include question boxes for respondents to provide any feedback. Next, the revised CCI was administered to 30 patients. Many patients expressed confusion with the tense of questions; thus, the questions were revised to reflect present tense. Additionally, one question that puzzled nearly all patients was removed. Results of the third field test indicated that two questions had over 50% N/A (not applicable) response rates, suggesting that these questions may not be relevant to many cancer patients and were candidates for removal. Further, participants suggested that several questions may be improved by modifying wording to make them less concrete and more conceptual. For example, based on the feedback, a question “I feel that my providers address my nausea and vomiting” was revised to “I feel that my providers will address any concerns such as nausea and vomiting should I experience this” to improve applicability of the question to a broader cancer patient population. Additionally, in order to improve interpretability of responses, the N/A response option was dropped as this response option does not distinguish optimal versus suboptimal CC according to patients’ perspectives [47]. The “neutral” response option was retained.

**Fig. 1:**
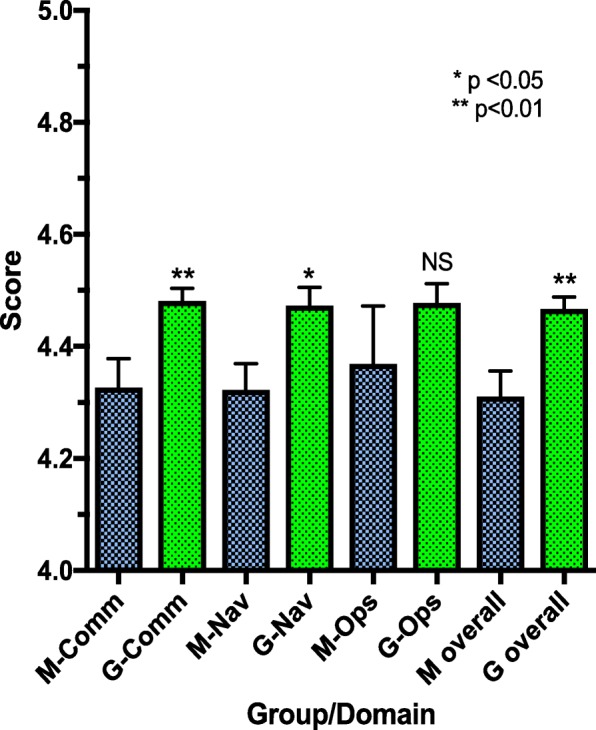
Mean score comparisons for overall and 3 domain scores by groups, M = Myeloma, G = GI

In the final field testing at Mount Sinai Hospital, the revised CCI was administered to 60 patients, 30 myeloma and 30 GI cancer patients from separate practices within the same academic hospital setting. Patients receiving care from the GI oncology practice reported significantly better perception of CC than those receiving care from the myeloma practice on Overall (*p* < .01), Communication (*p* < .01), and Navigation domain scores (*p* < .05). Consistent with expectations, there was no significant difference in the Operational domain scores between the two groups. Figure 1 illustrates the mean score comparison for overall and three domain scores between the two groups.

The initial version of the CCI following field testing consisted of 27 multiple-choice items rated on a 5-point Likert-scale from *Strongly Agree* to *Strongly Disagree,* with a response option “Not Applicable”. The Communication domain assesses information regarding the quality of communication among the various components of the health care team(s) and between the healthcare team and patients. The Navigation domain provides an assessment of patients’ perceptions about global aspects of care beyond the physician’s office or practice site such as information about financial resources, insurance, and social and educational issues. The Operational domain is a unique subscale in the CCI that probes for information regarding how a patient perceives his/her care is coordinated related to access to care, scheduling issues, and efficiency of care delivery. This instrument was designed for use with patients with any cancer type receiving active treatment and across varied healthcare settings. The three domains were developed based on feedback obtained through focus group discussions and cognitive interviews with patients and providers during field testing. Specifically, the Operational domain that is unique to the CCI was deemed as a critical component of CC by cancer patient participants in focus groups and cognitive interviews.

### Study 2: validation and refinement

Sixty-eight cancer patients on active therapy participated in seven focus group sessions held between February 2018 and August 2018. As can be seen in Table 1, the mean age of this sample was 61.8, and the majority of participants were female (78%). Participants were ethnically diverse with various cancer types, with a high proportion of women with breast cancer (see Table 1). The majority of participants indicated receiving care in a hospital-based facility (60%). Approximately one-fourth of the participants reported receiving care across multiple settings.

**Table 1:** Sample characteristics

	n (%)	M (SD)
Age (mean)		61.8 (12.05)
Female	53 (78)	
Male	14 (21)	
Race (any)		
American Indian/Alaska Native	2 (3)	
Filipino	10 (15)	
Japanese	16 (24)	
Native Hawaiian/Part Hawaiian	14 (21)	
Other Asian/Race	4 (6)	
White	25 (37)	
Multi-race/ethnicity	20 (29)	
Cancer Type		
Brain	1 (1)	
Blood	1 (1)	
Breast	38 (56)	
GI	2 (3)	
GYN	4 (6)	
Male	5 (7)	
Lymphoma	4 (6)	
Practice Setting		
Private practice	6 (9)	
Hospital-based	41 (60)	
Other/Both	17 (25)	

Based on the final coding, thematic analysis of the focus group transcripts was conducted. Analysis demonstrated the following most prevalent themes: Navigator, Team, Survey, and Communication. Other themes included Access, Insurance, Providers, Support, Advocacy, Education & Knowledge, Advocate, Facility, and Timing. These themes were developed by the research team following independent review of the focus group transcripts. Table 2 presents descriptions/subthemes for each theme and the number of focus groups in which each theme was discussed. Overall, patients expressed concordance with the CCI items and their care coordination experiences, thereby establishing face and content validity of the CCI.

**Table 2:** Themes and descriptions/subthemes and the number of focus groups in which each theme was discussed

Themes	Descriptions/Subthemes	n FGs
Navigator	Lack of navigator, navigator identification, information	7/7
Team	Team-based care, change in physician/health provider, second opinion, specialist	6/7
Survey	Survey structure, wording, survey format, global question	7/7
Communication	Communication among health providers/between patient and health provider(s)	7/7
Access	Access to treatment, availability of appointments	5/7
Insurance	Health insurance coverage, inadequate coverage, billing-related issues	4/7
Providers	Change in physician/health provider, second opinion, specialist	6/7
Support	Support services, emotional/financial/social support, support groups	6/7
Advocacy	Advocacy for treatment/information by family/friend/other	4/7
Education & Knowledge	Treatment information, patient education, resources, alternative medicine, clinical trials, nutritionist	6/7
Advocate	Need to serve as one’s own advocate, patient advocate providers, information delivery, electronic health records	3/7
Facility	Privacy, cleanliness of facility	3/7
Timing	Treatment stage, when navigator was assigned	1/7

As can be seen in Table 2, the highest proportion of focus group comments was related to the navigator theme. The majority of participants indicated that they did not have access to a patient navigator or were unfamiliar with the term “patient navigator”; thus, a higher than expected proportion of participants’ comments were related to the navigator theme. Regarding the theme on team-based care, many participants expressed confusion with the terms that referred to their care teams such as “my oncology team” or “member of my oncology team,” as they either did not have an oncology team or did not perceive receiving care from a team of providers. Relatedly, many focus group comments were related to survey wording regarding health provider(s). For example, some patients indicated that they did not see an oncologist, as their cancer care was provided by a urologist or another physician. Thus, questions with the wording “team” and “my oncologist” were revised to refer to treating physician(s) as “my cancer doctor” to increase relevancy to actual care. Regarding specific CCI intent, some suggestions from participants included re-wording of some items, use of more concrete/direct language, and an inclusion of a global question to assess overall perceptions of CC. Discussions regarding communication varied widely; some patients expressed challenges with communication with providers, whereas others had favorable experiences, in particular with use of electronic health records.

Participants had unique care coordination experiences, and these experiences were reflected in the comments provided in focus group discussions. Such comments broadly addressed access to treatment, insurance, providers, support, advocacy, education and knowledge. These comments provided additional insights regarding content validity as well as potential areas of refinement of the CCI. For example, patients expressed concordance with questions that probed regarding the availability of appointments and access to providers or their staff during and outside of normal business hours for any questions or complications from treatment. Insurance-related discussions unveiled some unexpected CC challenges that were not included in the initial CCI. The CCI question regarding information on support groups was described by most participants as essential as they were deemed socially and emotionally beneficial, and increased knowledge about the disease and treatment process provided by peers positively impacted patients’ experiences. Regarding advocacy, some participants emphasized the importance of the CCI questions on seeking a second opinion and other treatment-related resources on their own or having to rely on a family member/friend to provide support. Other less commonly discussed themes included advocate, facility issues, and treatment timing, all of which appeared in less than half of the focus groups.

Following qualitative analysis, results of the CCI completed by focus group participants were examined. Visual inspection of the responses revealed that some questions had limited variability or response ranges. For example, responses to some items such as “In general, I know exactly where to go for my appointments” were nearly unanimously “*Strongly Agree*”, indicating that these items did not have sufficient variability in responses; thus, these items were removed from the final CCI. Further, some items including “I was told what paperwork I needed to bring to my first appointment” had high proportions of missing, neutral, or not applicable written responses. These items were discussed and modified or removed by the research team. The response option “Neutral” was also removed, as responses to this category presented with multiple potential interpretations (e.g., not sure, not applicable, unknown) thereby reducing validity of the survey results. Furthermore, based on focus group suggestions, additional items were incorporated into the questionnaire. These new additional questions were designed to address insurance and financial aspects of cancer care, availability of family member/relative/friend help with care coordination, information about clinical trials, and a global question to assess overall perceptions of CC.

The final CCI questionnaire from Study 2 consists of 29 items rated on a 4-point Likert scale from *Strongly Disagree* to *Strongly Agree*. These items load on the three domains as follows: 16 Communication, 14 Navigation, and 10 Operational. Item-domain loadings were nominated by three members of the research team and subsequently finalized by consensus. Some items were specified to load on more than one domain, as some aspects of these domains overlap conceptually. Psychometric evaluation of the CCI version tested in the focus groups demonstrated that it has excellent overall internal consistency reliability, Cronbach’s α = .922. Internal consistency reliability for the three domains were adequate to excellent; Communication α = .916, Navigation α = .793, and Operational α = .738. Reading level analysis of the final CCI demonstrated acceptable reading level, Flesch-Kincaid Grade Level = 8.2.

## Discussion

Although the importance of coordinated care has been well-recognized, the paucity of measures to assess cancer care coordination limits efforts to improve cancer care delivery. As patient-centered care delivery research evolves, tools for assessment aimed at improving care delivery are needed. The present study used mixed-methods to develop, validate, and refine a new care coordination instrument, CCI, designed to assess cancer patients’ perception of care coordination across varied healthcare settings and populations. Overall, the results indicated that the CCI has excellent psychometric properties and is a useful tool to assess patients’ perception of cancer care coordination. Findings from qualitative analysis provided a rich, in-depth evaluation of patients’ care coordination experiences, with emerging themes that captured perceptions of cancer CC from diverse individual experiences. Knowledge gained from the use of this tool may provide important first-hand information from patients and identify specific CC processes and activities that may be targets for effective CC intervention efforts.

In the first part of this study, the instrument development process and initial field testing results were described. Questionnaire development is a complex process, and a number of iterative steps were used to test and further refine the instrument across multiple patient samples to improve clarity and increase relevance. Instrument development was informed by existing CC framework and measures, and the CCI was designed to include three domains conceptualized as critical components of CC: Communication, Navigation, and Operational. Expanding on the IOM model, the CCI’s Communication domain was conceptualized to assess communication among health providers as well as between patient and health providers, and it also includes assessment regarding whether patients’ preferences and opinions were incorporated as part of information provision. A departure from existing measures, the CCI includes a unique domain, an “Operational” subscale, that assesses patients’ views on access to care, scheduling, and efficiency. In the final field testing, preliminary findings of discriminant validity of the CCI domains were reported, as evidenced by expected and observed differences in mean scores between two groups receiving specialty care within the same hospital.

Consistent with prior studies, participants in the focus groups expressed various challenges associated with CC including communication and lack of informational resources [22, 28]. Focus group discussions revealed important themes regarding care coordination based on patients’ perspectives and some important distinctions between hypothesized CC based on the literature and actual CC. For example, although team-based approach to care is conceptualized as a gold standard for cancer care [3], many participants in this study did not perceive their care as delivered by a team of health care providers or a cancer care team. Further, despite the growing interest in a patient navigator approach, the majority of participants indicated that they did not have a patient navigator or were unfamiliar with patient navigator programs or resources. These contrasting views may be in part due to prior research focused on systems and providers’ perspectives rather than patients’ [15–19]. The current findings suggest the need to expand cancer CC research to include broader samples of cancer patients receiving care in private, community-based, and other hospital settings.

There are many strengths to the present study. First, this study contributes a new patient-centered instrument for assessment of cancer CC in varied care settings and populations. Expanding on existing CC framework and measures, the CCI has a unique domain, “Operational”, which has not been found in other CC measures. This new domain is conceptualized to assess patients’ views related to access to care and efficiency, and it provides an additional dimension to capture specific CC processes and activities important to patient-centered care. Second, based on the results, the CCI has excellent psychometric properties with internal consistency and good face and content validity. Given that rigorous psychometric evaluations of existing measures designed for US cancer patient population are currently lacking [35], the CCI presents as a promising new tool. Third, the CCI items were modified and refined according to feedback from patients receiving care in an academic setting as well as those receiving community-based care in varied health care settings, increasing generalizability of the CCI to “real-life” cancer care. Importantly, this study has illuminated notable differences between the existing literature and actual CC approaches used in cancer care. Although a multidisciplinary, team-based approach to cancer care in the US is extensively studied and recommended [3, 4, 8, 25], patient-reports of actual care indicated that many do not perceive receiving a team-based care and some patients receive oncology care from non-oncology providers (e.g., urologists, primary care). It is possible that these differences are in part due to that many prior studies on cancer CC were based on systems or providers’ perspectives of CC or focused on specific systems of care. Thus, these and other insights gained from this study provide important groundwork for further research in cancer care coordination.

Conversely, there are several limitations to the present study. First, the study samples were relatively small and comprised primarily of female patients and patients with breast cancer; thus, current findings are preliminary and need further replication in a larger sample. Second, given the nature of the focus group research, participants in Study 2 were limited to those who had volunteered to attend the focus group session at the research sites, were mobile, and had access to transportation. Cancer patients who are inpatient, have difficulty with transportation or mobility, or not well enough to participate in focus groups discussions were not part of this study. It is possible that patients with more advanced stage of the disease, receiving inpatient care, or in poorer health may have additional CC challenges beyond the scope of this study. Third, although the present study utilized patient samples receiving care in academic hospital and community-based settings, it is unknown how the CCI may generalize to closed systems such as Kaiser and Intermountatin systems [48, 49]. Future research will include validation of the CCI in closed systems.

### Implications

The present work has many applied and research implications for improving cancer care delivery. As indicated by the IOM’s landmark report on cancer care [3], patient-centered care that incorporates patients’ preferences and experiences requires informed and participatory patients and families. To that end, an instrument with adequate psychometric properties that assesses patients’ perspectives of care coordination is needed to further our understanding of current CC approaches, assess quality of CC, and provide valuable information regarding variables associated with optimal and poorly coordinated care. Such a tool is critical for healthcare professionals and entities to identify potential targets for CC improvement, monitor variations over time, and develop and shape intervention efforts designed to enhance healthcare delivery. Given that lack of rigorous psychometric evaluation of existing measures is one of the current limitations in cancer measures [35], this mixed-methods research to develop and validate a new care coordination instrument is an important first step to establishing an evidence-based, psychometrically sound patient-centered CC measure. The present study expands on the existing literature on cancer measures and provides a novel tool that can be used across varied care settings and populations. Future research using a larger sample will confirm the factor structure and psychometric properties of the CCI and demonstrate the utility of the instrument in a variety of different practice settings and patient populations.

Interestingly, the results of focus group interviews suggest that the existing literature and patient-reports of “real-life” care offer contrasting views of some aspects of cancer CC. These findings underscore the need for further investigation of current CC approaches used in cancer care and incorporation of patients’ perspectives when developing interventions to improve cancer care coordination. Additionally, subgroups of cancer patient populations such as racial and ethnic minority groups experience poorer cancer health outcomes, in part due to poorly coordinated care [30, 31]. The CCI, which has been refined in a racially diverse sample of cancer patients, may be particularly useful in efforts to further understand cancer CC and reduce cancer health disparities across specific race/ethnic populations. An important direction for future research is to examine utility of the CCI in African American and Latino cancer patients.

## Conclusions

Improving cancer care coordination is a priority. Many gaps in the literature including lack of adequate measures to assess patients’ perspectives of cancer care coordination hamper current efforts to improve cancer care delivery. A patient-centered approach to cancer care requires a psychometrically sound measure to assess care coordination from patients’ perspectives. Overall, the current findings are encouraging in that the new instrument, CCI, has demonstrated excellent reliability and validity in both academic and community-based samples. Use of this tool in applied clinical settings may provide health care providers and health care entities with capabilities to assess optimal CC strategies, monitor the effectiveness of CC intervention, and identify ways to improve cancer care delivery for all cancer patients.

## Supplementary information

**Additional file 1.** Focus group discussion guide.

## Data Availability

The datasets used and/or analyzed during the current study are available from the corresponding author on reasonable request. Any information made available will be fully de-identified.

## Abbreviations

AHRQ: Agency for Healthcare Research and Quality
CAHPS: Consumer Assessment of Healthcare Providers and Systems
CC: Care Coordination
CCCP-Q: Cancer Care Coordination Questionnaire for Patients
CCI: Care Coordination Instrument
GI: Gastrointestinal
IOM: Institute of Medicine
NP: Nurse Practitioner
RN: Registered Nurse
