# Supplementary material for: Development and psychometric evaluation of a questionnaire to measure cancer patients’ perception of care coordination
Source: BMC Health Serv Res. 2020 Jan 21;20:52. doi: 10.1186/s12913-020-4905-4 (PMC6975072; doi:10.1186/s12913-020-4905-4)
Supplement: Supplementary file 1 — Additional file 1. Focus group discussion guide. [file 12913_2020_4905_MOESM1_ESM.docx]

Supplementary File. *Focus Group Discussion Guide.*

| Key Questions |
| --- |
| 1. What did you think about the survey? |
| 2. Which questions were not clear or confusing? If so, how? |
| 3. How did this survey capture your experiences with care coordination? |
| 4. What information related to care coordination that you think might be missing from the current survey? |
| 5. What are your thoughts about completing this survey on a paper like this survey or in an online format like on a smart phone? |
| Ending Questions |
| 1. We wanted you to help us evaluate the survey. The purpose of the survey is to measure cancer patients’ perspectives of care coordination. We wanted to know how to improve the survey and whether this survey adequately captures cancer patients’ experiences with care coordination. |
| 2. Is there anything that we missed? |
| 3. 3. Is there anything that you might have wanted to say that you didn’t get a chance to say? |
